# From Abroad to Appalachia: A case report of cutaneous leishmaniasis in central Pennsylvania

**DOI:** 10.1016/j.jdcr.2025.09.032

**Published:** 2025-10-10

**Authors:** Natasha Salmen, Rebecca Metellus, Olivia Lim, Arjun Pandya, Mikael Horissian

**Affiliations:** a Northeast Ohio Medical University, Rootstown, Ohio; b Geisinger Commonwealth School of Medicine, Scranton, Pennsylvania; c Marshall University, Huntington, West Virginia; d Geisinger Health, Department of Dermatology, Danville, Pennsylvania

**Keywords:** cutaneous leishmania (CL), epidemiology, leishmaniasis, tropical disease

## Introduction

Cutaneous leishmaniasis (CL) is a tropical disease caused by protozoa of the genus *Leishmania* and is transmitted through the bite of a female sandfly during a blood meal.^1^ Depending on the species and host response, infection may lead to cutaneous, mucocutaneous or visceral leishmaniasis. This condition occurs globally and is classified based on geographic location. New World leishmaniasis is found in the Western Hemisphere from the southern United States to South America. In contrast, Old World leishmaniasis is prevalent in the Eastern Hemisphere, including Southern Europe, Asia, and Africa.^2^

CL is more prevalent in the Old World, with 1.5 to 2 million new cases reported annually.^3^ In uncomplicated CL cases, the parasites remain confined to the skin, causing chronic, slow-healing ulcers. These painless lesions pose no immediate threat to life and can resolve without treatment. Healing may take several months and often results in disfiguring scars.^4^ This case report highlights a rare presentation of CL in the northeastern United States.

## Case report

**Fig 1:**
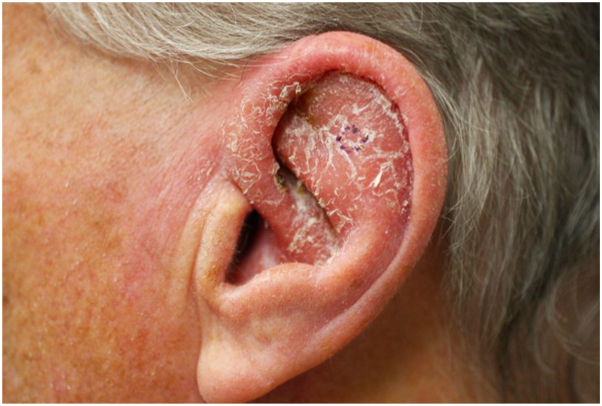
Erythematous and edematous plaque with superficial scale and crust encompassing entire left ear, sparing the earlobe.

**Fig 2:**
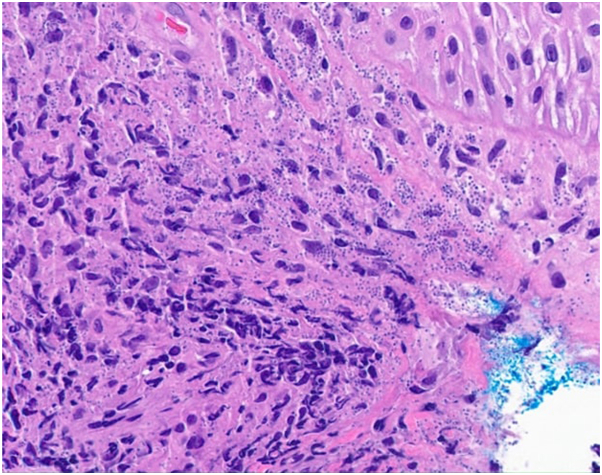
H&E demonstrating dense infiltrate composed of histiocytes with intracellular amastigotes.

**Fig 3:**
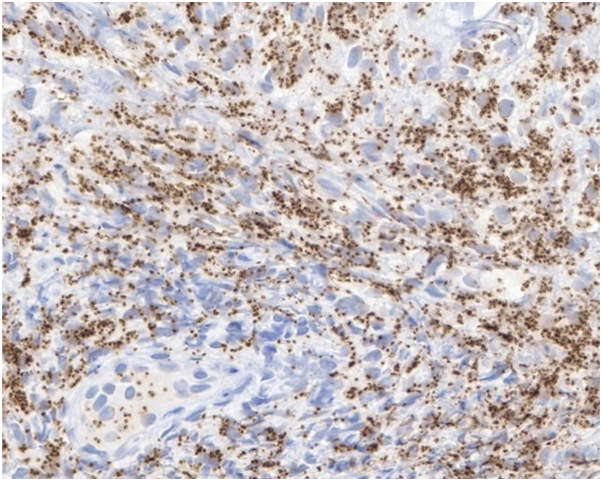
CD1a stain highlighting intracellular amastigotes brown.

An 80-year-old male presented to outpatient dermatology for evaluation of a persistent pink scaly rash on his left ear that initially developed 1.5 years ago as a crusted papule. Over the next few months, this crusted papule slowly began to turn into a scaly pink plaque with diffuse erythema and edema encompassing the cartilaginous portion of the left ear, adjacent preauricular and retroauricular skin, and sparing the earlobe (Fig 1). He did not recall any trauma or bites, and he further denied any bleeding, ulceration, fever, chills, weight loss, and fatigue. Before the onset of this rash, the patient had traveled extensively to tropical locations, including Cancun, Mexico; Mallorca, Spain; and Antigua. Oral antibiotics and intramuscular steroids were previously used to treat the rash with minimal improvement. A biopsy was performed and revealed numerous macrophages and histiocytes with phagocytized amastigotes (Fig 2). A confirmatory stain with Cd1a was positive, leading to a diagnosis of CL (Fig 3).

**Fig 4:**
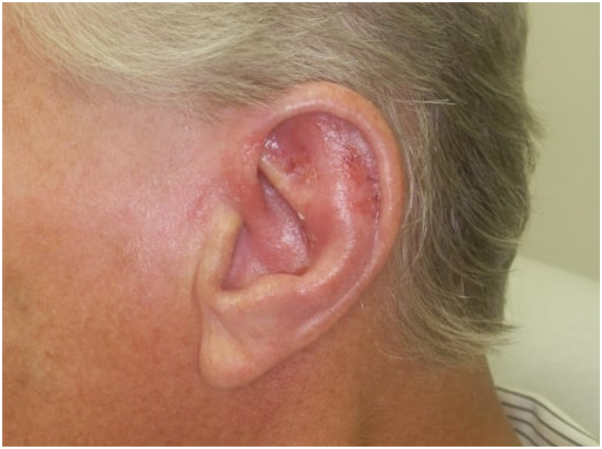
Follow-up image of patient’s left ear s/p topical paromomycin and oral miltefosine with significantly improved erythema and edema.

Infectious disease was consulted, and a punch biopsy was performed and sent for polymerase chain reaction for further speciation, which confirmed Leishmania donovani/infantum/chagasi species complex. Confirmatory rapid kala-azar serum test was negative for visceral disease. Following guidance from the CDC and infectious disease, the patient was prescribed topical paromomycin 15% to apply once daily for 20 days. At the 6-week follow-up appointment, there was minimal improvement from the paromomycin therapy, and the rash persisted. Systemic therapy with Miltefosine 50 mg 3 times daily for 28 days was initiated with close laboratory monitoring. Two weeks after completing Miltefosine, the patient demonstrated a significant improvement in his cutaneous rash. Edema, erythema, and scaling also improved (Fig 4). The patient’s next follow-up is scheduled for 4 weeks.

## Discussion

Factors such as increased international travel, migration, and climate change have likely contributed to the distribution of Leishmaniasis in nonendemic areas.

### Dissemination in nonendemic regions

CL is often travel-related, with tourism, military service, or work in endemic areas increasing the infection risk. A European retrospective study found that 62% of imported CL cases were from the Old World and 38% from the New World.^5^ Individuals migrating from endemic areas may also present with CL. While CL is rare in nonendemic regions, rising travel contributes to its spread. Since no vaccine exists, travelers should be advised to use insect repellent and wear protective clothing to prevent sandfly bites. Likewise, physicians in nonendemic areas should consider CL in the differential diagnosis for patients with an extensive travel history.

### Climate change

Climate change is believed to contribute to the emergence of CL in nonendemic regions. Rising temperatures in cooler areas may enable the survival of sandflies, facilitating the spread of leishmaniasis.^6^ Additionally, one study predicts that leishmaniasis will gradually expand across the United States and potentially into Canada, with the number of individuals at risk expected to double by 2080.^7^

### Zoonosis

In addition to expanding into new regions, leishmaniasis may find new hosts for transmission. The life cycle begins when an infected sandfly, the invertebrate vector, bites a vertebrate host – either an animal or a human – releasing the parasite into the skin, where it replicates and triggers an immune response. Various vertebrate hosts known to carry *Leishmania* have been identified in endemic areas, but the spread of sandflies to new regions could introduce new host species.^6^

### Immune response

Upon transmission, the parasite becomes phagocytized by macrophages. *Leishmania* also evades macrophage destruction by resisting reactive oxygen species and modulating intracellular signaling. Dendritic cells present *Leishmania* antigens to activate the adaptive immune response, a crucial step in determining disease outcome. A protective Th1 response, marked by interferon gamma and tumor necrosis factor alpha production, activates macrophages to produce nitric oxide, an essential compound for killing parasites. In contrast, a dominant Th2 response, marked by interleukin-4, interleukin-5, and interleukin-13, suppresses macrophage activation and facilitates parasite persistence. Certain *Leishmania* species, like *L. major,* are effectively controlled by a Th1 response, whereas others, like *L. donovani*, are more prone to immune evasion and chronic infection. Coinfections, malnutrition, and immunosuppression further impact disease progression.^8^

### Treatment

Treatment of leishmaniasis depends on the degree of involvement. Patients with limited cutaneous disease may be treated with intralesional or topical paromomycin with or without topical gentamicin (aminoglycosides). This has demonstrated favorable results and clearance in over 80% of patients.^9^ An anatomic site like the ear, compounded with possible chondritis, may pose a treatment challenge due to the lack of robust blood supply to the cartilage, leading to lower antibiotic absorption. Patients with visceral disease or cutaneous disease recalcitrant to topical treatments may require a more aggressive approach with intravenous or oral medications such as miltefosine, amphotericin B, sodium stibogluconate, or pentamidine.^10^

## Conclusion

CL is a rare condition infrequently encountered in nonendemic regions like central Pennsylvania. Due to increased travel and climate change, physicians in these areas should maintain a high index of clinical suspicion for vector-borne diseases, such as CL. The CDC is a vital resource which may guide diagnosis and management for this condition.

## Conflicts of interest

Dr Horissian is a speaker for Union Chimique Belge and past consultant for Bristol Myers Squibb. Authors Salmen, Metellus, and Drs Lim, and Pandya have no conflicts of interest to disclose.
